# Identifying essential proteins from active PPI networks constructed with dynamic gene expression

**DOI:** 10.1186/1471-2164-16-S3-S1

**Published:** 2015-01-29

**Authors:** Qianghua Xiao, Jianxin Wang, Xiaoqing Peng, Fang-xiang Wu, Yi Pan

**Affiliations:** 1 School of Information Science and Engineering, Central South University, 410083 Changsha, China; 2 School of Mathematics and Physics, University of South China, 421001 HengYang, China; 3 Division of Biomedical Engineering, University of Saskatchewan, Saskatoon, S7N 5A9 SK, Canada; 4 Department of Computer Science, Georgia State University, 30302-4110 Atlanta, USA

**Keywords:** Essential proteins, Protein-protein interaction, Dynamic gene expression profiles, Active protein-protein interaction networks, Centrality measures

## Abstract

Essential proteins are vitally important for cellular survival and development, and identifying essential proteins is very meaningful research work in the post-genome era. Rapid increase of available protein-protein interaction (PPI) data has made it possible to detect protein essentiality at the network level. A series of centrality measures have been proposed to discover essential proteins based on the PPI networks. However, the PPI data obtained from large scale, high-throughput experiments generally contain false positives. It is insufficient to use original PPI data to identify essential proteins. How to improve the accuracy, has become the focus of identifying essential proteins. In this paper, we proposed a framework for identifying essential proteins from active PPI networks constructed with dynamic gene expression. Firstly, we process the dynamic gene expression profiles by using time-dependent model and time-independent model. Secondly, we construct an active PPI network based on co-expressed genes. Lastly, we apply six classical centrality measures in the active PPI network. For the purpose of comparison, other prediction methods are also performed to identify essential proteins based on the active PPI network. The experimental results on yeast network show that identifying essential proteins based on the active PPI network can improve the performance of centrality measures considerably in terms of the number of identified essential proteins and identification accuracy. At the same time, the results also indicate that most of essential proteins are active.

## Introduction

Essential proteins play a decisive role in the survival and development of the cell. The identification of essential proteins is crucial to understanding the minimal requirements for cellular life and for practical purpose, such as drug design [1]. The prediction and discovery of essential genes have been performed by experimental procedures such as single gene knockouts [2], RNA interference [3] and conditional knockouts [4], but these techniques require a large investment of time and resources and they are not always feasible. Considering these experimental constraints, a highly accurate computation approach for identify essential proteins would be of great value. At the present, there are many computational approaches for predicting essential proteins based on their properties. Most of these research approaches focused on their topological properties in biological networks, such as protein-protein interaction (PPI) networks. Recently, many methods were proposed for detecting essential proteins based on network topology, such as degree centrality(DC) [5], betweenness centrality (BC) [6], closeness centrality (CC) [7], subgraph centrality (SC) [8], eigenvector centrality (EC) [9], information centrality (IC) [10], edge clustering coefficient centrality (NC) [11], local average connectivity centrality (LAC) [12], etc. These centrality measures were used to identify essential proteins based on network topology. Experiment results shown that they are better than pseudorandom selection in detecting essential proteins. However, there exist some limitations on these methods. The PPI data generated by high-throughput technologies is incomplete and contains many false positives and false negatives, which impacts the correctness of predicting essential proteins.

He et al. illustrated that some PPIs are more important than others in reality [13]. Some research works shown that many essential proteins have low connectivity and are difficult to be identified by centrality measures [13-16]. Many research works focused on identification essential proteins by integration PPI networks and other biological information, such as cellular localization, gene annotation, genome sequence, and so on [13,16,17]. Acencio et al. demonstrated that network topological features, cellular localization and biological process information are extremely useful for reliable prediction of essential genes [17]. Hart et al. pointed out that essentiality is a product of the protein complex rather than the individual protein [18]. Tew et al. [19] incorporated function information with topological information to detect essential proteins. Li et al. [20] proposed a new method to identify essential proteins by integration of PPI network topology with protein complexes information. Recently, Li et al. proposed a new method for predicting essential proteins based on the integration of PPI network and gene expression profiles [21], named PeC. Peng et al. [22] proposed an iteration method for predicting essential proteins by integrating the orthology with PPI networks. The current centrality measures were based on the topology of PPI networks. However, PPI network are static, which cannot reflect the real interaction in networks. In other words, the PPI data generated by high-throughput technologies is incomplete and contains many false positives and false negatives, which impacts the correctness of predicting essential proteins. In this paper, we propose a new method for predicting essential proteins based on active PPI network. We construct an active PPI network based on static PPI network and dynamic gene expression data. Then some centrality measures (DC, LAC, NC, BC, CC and SC) which are based on network topology have been applied to predict essential proteins based on the constructed active network. The experimental results show that it is more effective to predict essential proteins based on the active PPI network than based on static PPI network.

## Methods

In this section, we first construct an active PPI network based on dynamic gene expression profiles and static PPI network. Then, we identify essential proteins based on the constructed active PPI network.

### Time-dependent model and Time-independent model

Let *x *= {*x*_1_,..., *x_m_*,..., *x_M_*} be a time series of observation values at equally-spaced time points from a dynamic system. Wu et al. [23] have adopted AR (autoregressive) model to analyze the time dependence of time-course (dynamic) gene expression profiles. In [26], the time-dependent relationships can be modeled by an AR model of order *p*, denoted by *AR*(*p*), as follow:

(1) $$x_{m}=\beta_{0}+\beta_{1}x_{m-1}+\beta_{2}x_{m-2}+...+\beta_{p}x_{m-p}+\varepsilon_{m};m=p+1,...,M$$

where *β_i _*(*i *= 0, 1,..., *p*) are the autoregressive coefficients, and *ε_m_*(*m *= *p *+ 1,..., *M *) represent random errors, which independently and identically follow a normal distribution with the mean of 0 and the variance of *σ*^2^. The system of Model (1) can be rewritten in the matrix form as:

(2) Y=Xβ+ε,

where

$$Y=\left[\begin{array}{c} x_{p+1}\\ x_{p+2}\\ \vdots \\ x_{M} \end{array}\right],X=\left[\begin{array}{cccc} 1 & x_{1} & \cdots \; & x_{p}\\ 1 & x_{2} & \cdots \; & x_{p+1}\\ 1 & \vdots & \ddots & \vdots \\ 1 & x_{M-p} & \cdots \; & x_{M-1} \end{array}\right],\beta =\left[\begin{array}{c} \beta_{0}\\ \beta_{1}\\ \vdots \\ \beta_{p} \end{array}\right],\varepsilon =\left[\begin{array}{c} \varepsilon_{p+1}\\ \varepsilon_{p+2}\\ \vdots \\ \varepsilon_{M} \end{array}\right]$$

The likelihood function for Model (2) is

(3) $$L\left(\beta ,\sigma^{2}\right)={\left(2\pi \sigma^{2}\right)}^{-\left(M-p\right)/2}\text{exp}\left[-\frac{1}{2\sigma^{2}}||Y-X\beta |{|}^{2}\right].$$

If the rank (*X*) = *p *+ 1 holds, the maximum likelihood estimates of *β *and *σ*^2 ^are

(4) $$\hat{\beta }={\left(X^{T}X\right)}^{-1}X^{T}Y$$

and

$${\hat{\sigma }}^{2}={∥Y-X\hat{\beta }∥}^{2}/\left(M-p\right).$$

The value of the maximum likelihood is given by

(6) $$L\left(\hat{\beta },{\hat{\sigma }}^{2}\right)={\left(2\pi {\hat{\sigma }}^{2}\right)}^{-\left(M-p\right)/2}e^{-\left(M-p\right)/2}.$$

In Model (2), the matrix *X *has *p *+ 1 columns and *M *− *p *rows. Thus a necessary condition for *rank*(*X*) = *p *+ 1 is *M *− *p *≥ *p *+ 1 or *p *≤ (*M *− 1)/2.

On the other hand, the time-independent model is also an autoregressive model with the order of zero. That is a noisy profile can be modeled by

(7) $$x_{m}=\beta_{0}+\varepsilon_{m},m=p...,M,$$

where *β*_0 _is a constant number and *ε_m_*(*m *= *p*,...,*M *) are the random errors which are subject to a normal distribution independent of time with the mean of 0 and the variance of $\sigma_{c}^{2}$. The likelihood function for Model (7) is

(8) $$L\left(\beta_{0},\;\sigma_{c}^{2}\right)={\left(2\pi \sigma_{c}^{2}\right)}^{-\left(M-p\right)/2}\text{exp}\left[-\frac{1}{2\sigma_{c}^{2}}\overset{M}{\underset{m=p+1}{\sum }}{\left(x_{m}-\beta_{0}\right)}^{2}\right].$$

The maximum likelihood estimates of *β*_0 _and $\sigma_{c}^{2}$ are

(9) $${\hat{\beta }}_{0}=\frac{1}{M-p}\overset{M}{\underset{m=p+1}{\sum }}x_{m}$$

and

(10) $${\hat{\sigma }}_{c}^{2}=\frac{1}{\left(M-p\right)}\overset{M}{\underset{m=p+1}{\sum }}{\left(x_{m}-{\hat{\beta }}_{0}\right)}^{2}$$

respectively. The maximum values of the likelihood is given by

(11) $$L\left({\hat{\beta }}_{c},\;{\left({\hat{\sigma }}_{c}\right)}^{2}\right)={\left(2\pi {\left({\hat{\sigma }}_{c}\right)}^{2}\right)}^{-\left(M-p\right)/2}e^{-\left(M-p\right)/2},$$

where ${\hat{\beta }}_{c}$ is a (*p *+ 1) dimensional vector whose first component is ${\hat{\beta }}_{0}$ and others are zeros.

The likelihood ratio of Model (7) to Model (1) is given by

(12) $$\wedge =\frac{L\left({\hat{\beta }}_{c},{\left({\hat{\sigma }}_{c}\right)}^{2}\right)}{L\left(\hat{\beta },{\left(\hat{\sigma }\right)}^{2}\right)}={\left(\frac{{\left(\hat{\sigma }\right)}^{2}}{{\left({\hat{\sigma }}_{c}\right)}^{2}}\right)}^{\left(M-p\right)/2}$$

According to the likelihood principle, if Λ in Formula (12) is too small, the series *x *= {*x*_1_,..., *x_m_*,..., *x_M_*} is more likely time-dependent than time-independent. The statistic

(13) $$F=\frac{M-2p-1}{p}\left(\Lambda^{-2/\left(M-p\right)}-1\right)=\frac{M-2p-1}{p}\left(\frac{{\hat{\sigma }}_{c}^{2}}{{\hat{\sigma }}^{2}}-1\right)$$

follows an *F *distribution with (*p*, *M *− 2*p *− 1) degrees of freedom when Model (7) is true for a series of observations. When *F *is very large, thus the *p*-value is very small, Model (7) is rejected, i.e., observation series *x *= {*x*_1_,..., *x_m_*,..., *x_M_*} is time-dependent. From Formula (13), one can calculate the probability (*p*-value) that a series of observations is not time-independent. As the regression degree in Model (1) is unknown, the *p*-values are calculated by Formula (13) for all possible orders *p *(1 ≤ *p *≤ (*M *− 1)/2). The proposed method calls a gene to be significantly expressed (time-dependent) if one of these *p*-values calculated from its expression profile is smaller than a user-preset threshold value.

### Construction of the active protein interaction network

Tang et al. [24] use a potential threshold to filter noisy gene expression data, then construct an active PPI network. In their method the common value of a threshold is applied to all the genes and time points. Wang et al. [25] propose a method to identify active time points for each protein in a cellular process or cycle using a 3-sigma principle to compute an active threshold for each gene according to the characteristics of its expression curve, then construct an active PPI network. We first filter noisy genes based on time-dependent model and time-independent model, time-dependent genes expression data is more likely dynamically deterministic than random while time-independent genes expression data is more likely random than dynamically deterministic. Those gene expression data are considered to be noises if they are time-independent and their means are very small. We then use a threshold function to compute an active threshold for each gene according to their expression data. We finally construct an active PPI network (NF-APIN) [26]. Our threshold function is described as follows:

(14) Active_threshold=u+kσ×(1-F)

(15) $$F=\frac{1}{1+\sigma^{2}}$$

For each gene, *u *and *σ *are the mean and standard deviation of its expression values. The *Active threshold *is calculated by Formula (14) for all possible values *k*(0 ≤ *k *≤ 3). In this paper the value of coefficient *k *is selected as 2.5. If the expression level of a gene is over its active threshold at a time point, the corresponding protein is regarded as active at the time point. For each time point, if two proteins interacted with each other in the static PPI network are active at the same time point, the proteins and their interaction form a part of NF-APIN at the time point. The process is repeated until the NF-APIN is created.

### Centrality measures

A PPI network is usually regarded as an undirected graph *G *= (*V*, *E*), where a node *v *∈ *V *represents a protein and an edge *e*(*u*, *v*) ∈ *E *denotes an interaction between two proteins *v *and *u*. In our paper, we have described the active PPI network constructed by our strategy as *G' *= (*V'*, *E'*), a node *v *∈ *V' *represents a protein and an edge *e*(*u*, *v*) ∈ *E' *denotes an interaction between two proteins *v *and *u*. We assign *N *as the total number of nodes in the network. In graph theory and network analysis, centrality of a vertex measures its relative importance within a graph. At the present, six classical centrality measures based on network topology are defined as follows:

Degree Centrality (DC). The degree centrality of a vertex v is defined as

(16) DC(v)=deg(v)

Where deg(*v*) is degree of vertex *v*.

Betweenness Centrality (BC). The betweenness centrality of a vertex *v *is defined as the fraction of shortest paths that pass through the node *v*.

(17) $$BC\left(v\right)=\underset{s\neq v\neq t\in V}{\sum }\frac{\sigma_{st}\left(v\right)}{\sigma_{st}}$$

Where *σ_st _*is the total number of shortest paths from node *s *to node *t*, *σ_st_*(v) is the number of those paths that pass through *v*.

Closeness Centrality (CC). The closeness centrality of a vertex *v *is the reciprocal of the sum of graph-theoretic distances from the node v to all other nodes in the graph *G*.

(18) $$CC\left(v\right)=\frac{N-1}{\sum_{v\neq u\in V}d\left(v,u\right)}$$

Where *d*(*u*, *v*) is a natural distance between all pairs of nodes, defined by the length of their shortest paths.

Subgraph Centrality (SC). The subgraph centrality of a vertex *i *is the total number of closed walks in which v takes part and gives more weight to closed walks of short lengths.

(19) $$SC\left(i\right)=\overset{\infty }{\underset{k=0}{\sum }}\frac{\mu_{k}\left(i\right)}{l!}=\overset{N}{\underset{j=1}{\sum }}{\left[v_{j}^{i}\right]}^{2}e^{\lambda_{j}}$$

where *µ_k_*(*i*) is the number of closed walks of length *l *starting and ending at protein *i*, *v*_1_, *v*_2_,...*v_N _*is an orthonormal basis of *R_N _*composed by eigenvectors of the adjacency matrix A of the network and *λ*_1_, *λ*_2_,...*λ_N _*are the corresponding eigenvalues. where $v_{j}^{i}$ denotes the *i*th component of *v_j_*.

Local Average Connectivity Centrality (LAC). The local average connectivity of a node *v *(LAC(*v*)) is defined as the average local connectivity of its neighbors:

(20) $$LAC\left(v\right)=\frac{\sum_{w\in N_{v}}deg^{C_{v}}\left(w\right)}{\left|N_{v}\right|}$$

where *N_v _*is the set of neighbors of node v, *C_v _*is the subgraph *G*[*N_v_*] besides *N_v_*. For a node *w *in *C_v_*, deg(*w*) is its degree.

Edge Clustering Coefficient (NC) [11]. The edge clustering coefficient of *E_u,v _*can be defined by the following expression:

(21) $$ECC\left(u,v\right)=\frac{Z_{u,v}}{mid\left(d_{u}-1,d_{v}-1\right)}$$

Where *Z_u,v _*denotes the number of triangles that include the edge actually in the network, *d_u _*and *d_u _*are degrees of nodes *u *and *v*, respectively.

## Results

### Experimental datasets

The yeast's PPI network (20101010) is downloaded from DIP [27]. We filtered the self-interactions and repeated ones in the original PPI network. As a result, the PPI network used in our experiment has 5093 proteins and 24743 interactions. The yeast's dynamic gene expression data comes from [28], includes 6, 777 gene products under 36 different time points. The 6, 777 gene products in the gene express profile cover 95% of the proteins in the PPI network. The list of essential proteins of yeast downloaded from the following databases: MIPS [29], SGD [30], SGDP [31] and DEG [32], which contains 1285 essential proteins. Within the 1285 essential protein, 1167 proteins present in PPI network.

### Compare with seven typical Centrality measure in different PPI networks

In order to validate the performance of the proposed strategy, we conduct a comparison between two different PPI networks applying seven typical centrality measures defined in last section to predict essential protein.

**Figure 1:**
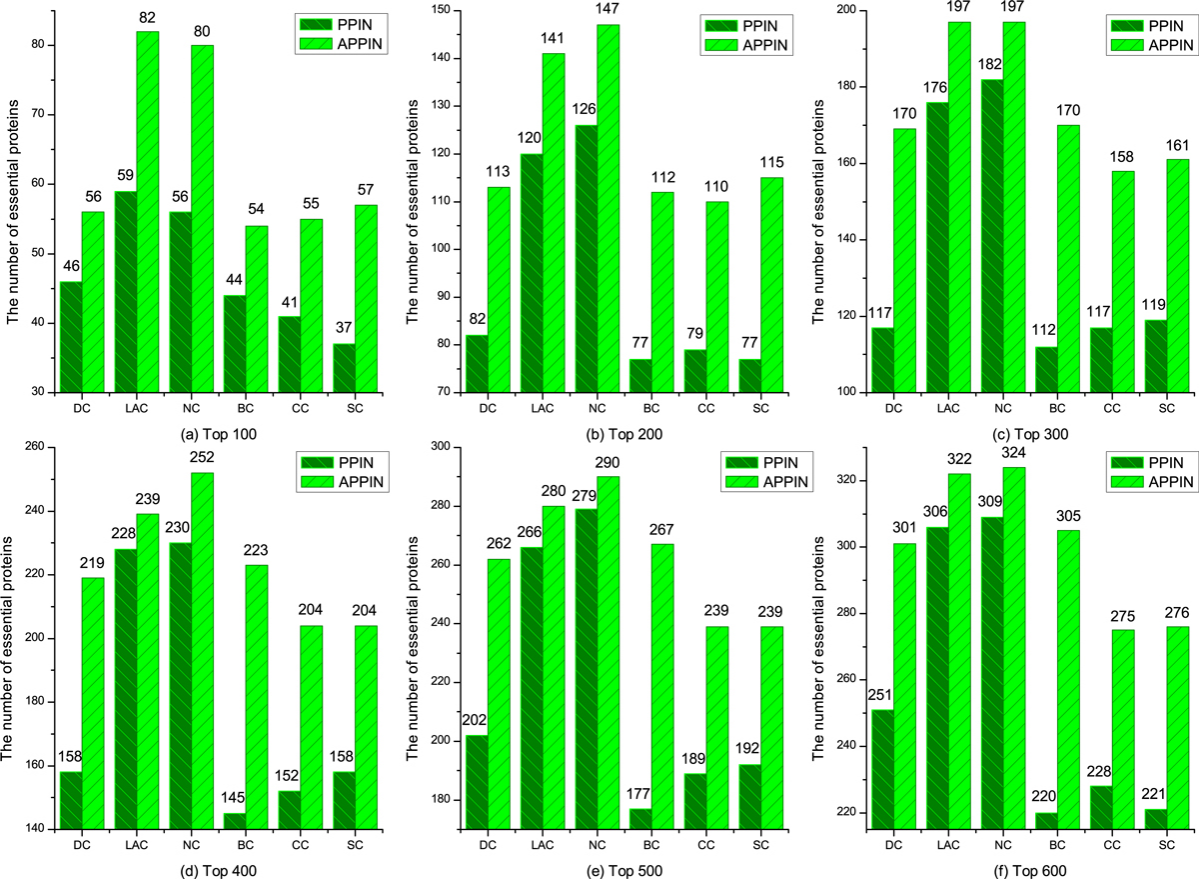
**Number of essential proteins detected by each methods in two different networks**. As is shown in Fig.1, the performance of each centrality measures in identifying essential proteins based on APPIN is better than PPIN. Especially, the improvements of SC based on APPIN are more than 50% when predicting 100 proteins, the number of essential proteins identified by LAC and NC based on APPIN achieves to 80.

Proteins are ranked in descending order according to their scores computed by each centrality measure. According to the sort, a certain number of top proteins should be regarded as essential proteins. With that, we select the top 100, top 200, top300, top400, top500 proteins as essential protein candidates and identify how many of these are true essential proteins. Numbers of essential proteins detected by seven typical centrality measures in two different networks are shown in Figure 1.

In Figure 1, PPIN denote that a certain centrality measure is applied based on the original PPI network of the yeast, and APPIN denote that a certain centrality measure is applied based on the active PPI network [24]. As is shown in Figure 1, the performance of each centrality measures in identifying essential proteins based on APPIN is better than PPIN. Especially, the improvements of SC based on APPIN are more than 50% when predicting 100 proteins, the number of essential proteins identified by LAC and NC based on APPIN achieves to 80.

**Figure 2:**
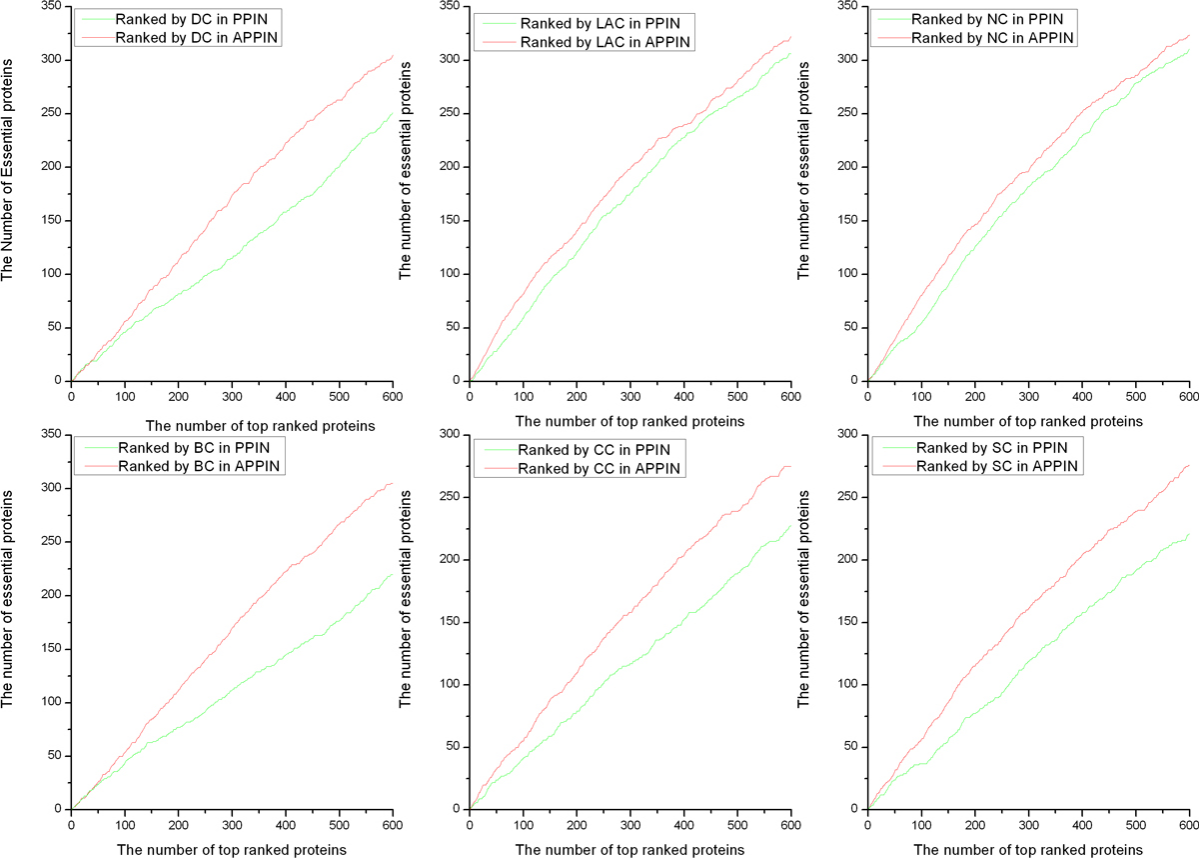
**DC, BC, CC, SC, LAC and NC are compared in two different networks by a jackknife methodology**. To further illustrate the efficiency of our strategy, we have analyzed by using a jackknife methodology. In Fig.2, proteins are ordered in descending according to their scores. The curve is plotted with the cumulative counters of true essential proteins and the cumulative counters of predicted essential proteins.

To further illustrate the efficiency of our strategy, we have analyzed by using a jackknife methodology [33]. In Figure 2, proteins are ordered in descending according to their scores. The curve is plotted with the cumulative counters of true essential proteins and the cumulative counters of predicted essential proteins. The areas under the curve (AUC) for each centrality measures in different networks are compared in Figure 2. It is obvious that the AUC for DC, BC, CC, SC, NC and LAC based on APPIN are better than PPIN.

In addition, we also conduct a comparison of overlaps true essential proteins predicted by each centrality measure in different two networks. The numbers of true essential proteins in top 100 predicted proteins are shown in Table 1 where *S*1 and *S*2 are the number of essential protein predicted in two different networks, respectively, *S*3 is the number of overlaps essential proteins. From Table 1 we can see that the number of common essential proteins identified in two networks is relatively low. This proves that identifying essential protein based on the active PPI network is a necessary complement. In conclusion, the efficiency of identifying essential proteins based on an active PPI network is better than the origin PPI network. This indicates that active proteins more like to be essential proteins.

**Table 1:** The case of overlaps essential proteins in different two networks when predicting 100 proteins

Centrality measures	*S*1/*S*2	*S*3	*S*1 − *S*3	*S*2 − *S*3
Degree Centrality (DC)	56/46	26	30	20
Betweenness Centrality (BC)	54/44	23	31	21
Closeness Centrality (CC)	55/41	12	43	29
Subgraph Centrality(SC)	57/37	10	47	27
Edge Clustering Coefficient (NC)	80/56	26	54	30
Local Average Connectivity Centrality (LAC)	82/59	35	47	24

## Conclusion

At present, the prediction of essential proteins is still a hot topic in the post-genome era. Many researches for identifying essential proteins are based on entire PPI networks. However, the PPI data obtained from various kinds of experimental techniques and methods, which generally contain false positives. It is insufficient to use original PPI data to identify essential proteins. In this study, we first filtered noisy genes based on dynamic gene expression profiles, and then constructed an active PPI network. After that, we predicted essential proteins based on our constructed active PPI networks using seven typical centrality measures. The experimental results show that the precision of identifying essential proteins based on our active PPI network is obviously higher than based on the origin PPI network. One direction of our further work is to apply the other prediction methods based on active PPI networks and confirm whether essential proteins have active characteristics.

## Competing interests

The authors declare that they have no competing interests.

## Authors' contributions

QX and JW obtained the protein-protein interaction data, gene expression data and essential proteins, generated the prediction model and drafted the manuscript. QX and XP performed experimental comparison and evaluated the results. JW, QX, FW, YP initiate the study and write the manuscript. All authors have read and approved the final manuscript.

## Declarations

The publication costs for this article were funded by the corresponding author.

This article has been published as part of *BMC Genomics *Volume 16 Supplement 3, 2015: Selected articles from the 10th International Symposium on Bioinformatics Research and Applications (ISBRA-14): Genomics. The full contents of the supplement are available online at http://www.biomedcentral.com/bmcgenomics/supplements/16/S3.
